# The Role of the Glycemic Index and Glycemic Load in the Dietary Approach of Gestational Diabetes Mellitus

**DOI:** 10.3390/nu16030399

**Published:** 2024-01-30

**Authors:** Ioanna Mavroeidi, Aspasia Manta, Athina Asimakopoulou, Alexandros Syrigos, Stavroula A. Paschou, Efthimia Vlachaki, Constantinos Nastos, Sophia Kalantaridou, Melpomeni Peppa

**Affiliations:** 1 Endocrine Unit, 2nd Propaedeutic Department of Internal Medicine, Research Institute and Diabetes Center, Attikon University Hospital, School of Medicine, National and Kapodistrian University of Athens, 12461 Athens, Greece; ioamavro@med.uoa.gr (I.M.); aspamanta@med.uoa.gr (A.M.); 2 3rd Department of Internal Medicine, Sotiria General Hospital, 11527 Athens, Greece; athasimak@med.uoa.gr (A.A.); ksyrigos@med.uoa.gr (A.S.); 3 Endocrine Unit and Diabetes Center, Department of Clinical Therapeutics, Alexandra Hospital, School of Medicine, National and Kapodistrian University of Athens, 11528 Athens, Greece; spaschou@med.uoa.gr; 4 Hematological Laboratory, 2nd Department of Internal Medicine, Hippokrateion Hospital, Aristotle University, 54640 Thessaloniki, Greece; 5 3rd Department of Surgery, Attikon University Hospital, School of Medicine, National and Kapodistrian University of Athens, 12461 Athens, Greece; kosnastos@med.uoa.gr; 6 Department of Obstetrics and Gynecology, Attikon University Hospital, School of Medicine, National and Kapodistrian University of Athens, 12461 Athens, Greece; skalanta@med.uoa.gr

**Keywords:** gestational diabetes mellitus, diet, nutrition, carbohydrates, insulin resistance, glycemic load, glycemic index, obesity, pregnancy, gestational insulin resistance, gestational inflammation

## Abstract

Gestational diabetes mellitus (GDM) is a common metabolic disorder that often develops during pregnancy, characterized by glucose intolerance and insulin resistance (IR). To ensure the well-being of both the mother and the fetus, the body undergoes multiple metabolic and immunological changes that result in peripheral IR and, under certain hereditary or acquired abnormalities, GDM in predisposed women. The adverse short- and long-term effects of GDM impact both the mother and the fetus. Nutrition seems to play an important role to prevent GDM or improve its evolution. An emphasis has been given to the proportion of carbohydrates (CHO) relative to protein and lipids, as well as dietary patterns, in GDM. The effects of CHO on postprandial glucose concentrations are reflected in the glycemic index (GI) and glycemic load (GL). Diets rich in GI and GL may induce or exacerbate IR, whereas diets low in GI and GL appear to enhance insulin sensitivity and improve glycemic control. These positive outcomes may be attributed to direct interactions with insulin and glucose homeostasis or indirect effects through improved body composition and weight management. This comprehensive narrative review aims to explore the significance of nutrition, with a focus on the critical evaluation of GI and GL in the dietary management of women with GDM.

## 1. Introduction

Gestational diabetes mellitus (GDM) is one of the most common pregnancy complications, with a steadily increasing prevalence, in parallel to the global rise in type 2 diabetes mellitus (T2DM) and obesity. Approximately 15% of pregnancies worldwide are complicated by GDM [1].

**Figure 1:**
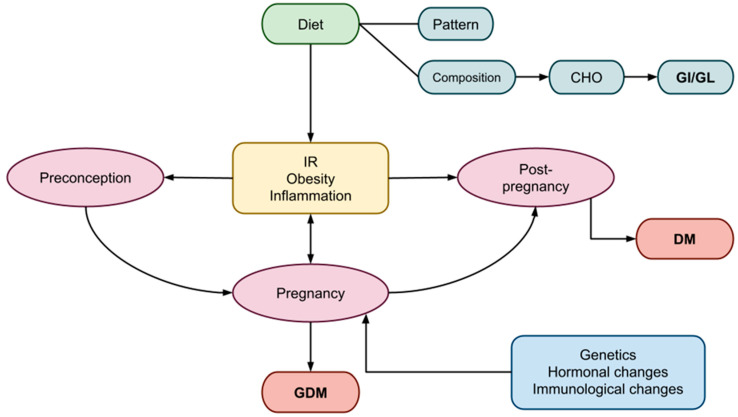
Factors influencing the periods of preconception, pregnancy, and post-pregnancy, leading to GDM. GDM, gestational diabetes mellitus; DM, diabetes mellitus; CHO, carbohydrates; GI, glycemic index; GL, glycemic load, IR; insulin resistance.

Pregnancy undergoes various anatomical, biochemical, physiological, and hormonal changes to meet the increased metabolic demands for fetal development. During early gestation, insulin sensitivity increases in order to control glucose metabolism and promote the uptake of glucose into adipose stores in preparation for the energy demands of mid- and late pregnancy. In adapting to ensure an adequate supply of carbohydrates (CHO) for the fetus and consequently maintaining the necessary glucose levels to meet its energy demands, a state of peripheral insulin resistance (IR) is gradually established [2]. As a result, blood glucose is slightly elevated across the placenta to fuel the growth of the fetus [3]. Thus, as the pregnancy progresses, a surge of hormones, including estrogen, progesterone, leptin, cortisol, placental lactogen, and placental growth hormones together promote a state of IR [3]. As part of the normal response, maternal tissues such as muscle and fat become relatively insulin-resistant. These maternal tissues increase the use of other fuel sources, such as fatty acids and ketone bodies [4]. Increased estrogen, progesterone, and insulin favor lipid deposition and the accumulation of maternal fat stores in early and mid-pregnancy and enhance fat mobilization in late pregnancy [5]. Protein catabolism is decreased as fat stores are used to provide energy for metabolism [6,7,8]. Hyperinsulinemia ensues as a compensatory response to the increased insulin production by β-cells as a means of compensating for the IR [1]. In addition to the hormonal imbalance, gestational IR is further exacerbated by genetic and epigenetic variables [9], increased visceral fat, altered gut microbiota [10], and obesity (Figure 1). Gestational IR is more evident in mid- to late pregnancy and typically resolves after delivery.

Also, a number of signaling pathways may play a role in the pathophysiology of GDM; NF-κB contributes to the development of GDM by promoting adipocyte inflammation and impairing insulin-related functions, such as glucose uptake, peroxisome proliferator-activated receptors (PPARs), sirtuins (SIRTs), 5′ AMP-activated protein kinase (AMPK), glycogen synthase kinase 3 (GSK3), PI3K/mTOR, inflammasomes, and the endoplasmic reticulum (ER) [11]. These key pathways often interact with and alongside each other, as T2DM studies have shown. Unfortunately, there is no clear evidence to indicate that these pathways act and contribute to GDM development [12]. Many biomolecules circulating in the blood or contained in the saliva have been studied as potential predictive markers in the diagnosis of GDM. In addition to adiponectin and leptin, these include galectins, growth differentiation factor-15, chemerin, omentin-1, osteocalcin, resistin, visfatin, vaspin, irisin, apelin, fatty acid-binding protein 4 (FABP4), fibroblast growth factor 21, and lipocalin-2 [13,14]. It has been found that high levels of FABP4 and low levels of irisin in the serum of pregnant women can be used as predictive markers. Levels of chemerin and resistin contained in the saliva are reported to be significantly higher in women with GDM than in healthy women [13].

A growing body of research highlights that hyperglycemia during pregnancy influences epigenetic processes, inducing changes through histone modification, DNA methylation, and the disrupted function of non-coding ribonucleic acid (ncRNA), including microRNAs (miRNAs) [15,16]. Mainly epigenetic changes lead to the dysregulation of gene transcription, modifying the phenotype of the developing child [17,18]. Short-term effects on offspring include respiratory distress syndrome, hypoglycemia, hyperbilirubinemia, hypocalcemia, hypomagnesemia, polycythemia, and adverse fetal programming, contributing to long-term risks including childhood obesity, T2DM, hypertension, and CVD in adolescence and adulthood [19,20,21]. Children of mothers with GDM or pregnant women with preexisting diabetes of any type (T1DM, T2DM, MODY, LADA, etc.) have an eight-times-higher risk of developing prediabetes or diabetes, compared to children of non-diabetic mothers [20]. Several studies report a high cardio-metabolic risk in children exposed to GDM in the womb, considering it an independent risk factor for glucose intolerance and CVD [11]. The risk of obesity (BMI ≥ 95th centile for age) is elevated in offspring exposed to gestational diabetes, type 1 diabetes, or type 2 diabetes in utero [22]. Also, the severity of diabetes (GDM requiring medications) during pregnancy may increase the vulnerability of offspring for depression or anxiety [23]. Preventing the epigenetic changes associated with metabolic and inflammatory processes in offspring underscores the critical importance of maintaining glucose control during GDM [17].

GDM is linked to complications for the mother as well, such as an elevated risk of GDM in future pregnancies, an increased risk of developing T2DM and early cardiovascular disease (CVD), as well as an increased risk of delivery through cesarean section [24].

Identifying modifiable risk factors for GDM prevention and management is, thus, crucial for the medium- and long-term well-being of both the mother and child [25]. Modifiable risk factors include maternal overweight or obesity, specific dietary choices, physical inactivity before or during pregnancy, and maintaining appropriate weight throughout pregnancy [19,26].

First-line therapies focus on major lifestyle adjustments, especially in nutrition. A balanced diet during pregnancy can keep gestational IR levels as low as possible to prevent GDM [24]. The timing and composition of food intake play a significant role, influencing circadian rhythms that regulate various physiological functions crucial to human health [27]. Food preparation might also produce advanced glycation end-products (AGEs), a major cause of oxidative stress in diabetes [28,29,30]. It is generally advised to consume macronutrients in moderation and balance while limiting your overall calorie intake [31].

The role of CHO has been extensively studied, with the type and amount affecting postprandial glucose levels and IR potential. A large number of dietary CHOs (glucose; sucrose; and cooked starches in pasta, potatoes, and white bread) are quickly digested and absorbed in the small intestine, causing a sharp increase in blood sugar levels. Higher-quality, nutrient-dense CHOs result in controlled fasting and postprandial glucose, as well as improved insulin action [21].

The glycemic index (GI) and glycemic load (GL) are measures proposed to assess CHOs’ effects on health in general and pregnancy, in particular [32,33]. The GI functions as an evaluative system for foods containing CHOs, illustrating the speed at which each consumed food impacts postprandial blood glucose levels. The GL, derived from the GI, is computed by multiplying the weight of available CHOs in the food (in grams) by the food’s GI and subsequently dividing by 100. In the context of glucose homeostasis, both indices precisely outline the type and quantity of CHOs present in a diet. By definition, foods with a high GI exhibit moderate to high levels of CHOs. Notably, certain items, such as fruits, whole grains in their natural state, and dairy products, also boast significant micronutrient contents [34]. High-GI and -GL diets have been associated with conditions such as obesity, diabetes, and CVD, all of which share IR as an underlying pathogenetic mechanism [25]. Conversely, meals with low GI and GL values contribute to the enhancement of insulin sensitivity and glucose homeostasis [18]. It is worth noting that in existing studies, a unanimous consensus on what defines a low GI/GL diet has yet to be reached. A list of basic foods with low GI and GL estimates is shown in Table 1 [25,35].

In this narrative review, we gathered available data on the effects of CHO diets, particularly GI/GL diets, on women with GDM. We aim to enhance our understanding of the optimal dietary recommendations for treating GDM, ensuring strict glycemic control while guaranteeing an adequate glucose supply for the fetus and addressing health issues affecting GDM patients and their offspring. The information utilized for this narrative review was collected following a comprehensive literature search using electronic databases.

## 2. Preconception

All women contemplating pregnancy, regardless of age, health status, and other risk factors, should be educated about the significance of adopting a healthy lifestyle and managing preexisting medical conditions prior to conception. Specifically, lifestyle interventions should be initiated approximately 6–12 months before conception to mitigate the risk of first-trimester miscarriage, perinatal mortality, and various other pregnancy-related complications [21,36,37].

Dietary habits, physical activity, and sleep cycles can impact ovarian quality, menstrual cycles, and ovulation. Conditions such as overweight, obesity, hypertension, T1DM, T2DM, hyperlipidemia, anemia, and vitamin deficiencies should be addressed and effectively managed before embarking on pregnancy [38].

Women who are underweight, overweight or obese, with known polycystic ovary syndrome (PCOS), or with poor eating habits are more prone to developing GDM [39]. These situations elevate the risk of IR and chronic inflammation, predisposing GDM to manifest earlier in pregnancy. Prospective observational studies have also discovered a link between prenatal high-GL diets and a higher incidence of GDM in US women [40]. Pre-pregnancy maternal obesity triggers a systemic inflammatory response [40,41,42], which may lead to downstream metabolic effects such as IR and glucose dysregulation, contributing to gestational hyperglycemia [41].

The early initiation of interventions, including dietary adjustments, exercise, and lifestyle counseling, can potentially reduce the risk of GDM [21]. Prior to conception, adopting a “healthy” diet, such as the Mediterranean diet or the DASH diet, has been linked to improved outcomes, particularly for obese and overweight women [43]. Preference is given to low-GI and -GL diets, especially when combined with plant-based proteins and fat, while caution is advised against low CHOs and high intake of animal-based products, as they may elevate the risk of GDM [44,45].

## 3. Pregnancy

### 3.1. Gestational IR

Insulin resistance is commonly observed during pregnancy, although the processes underlying its pathogenesis are complex and not yet fully understood [46]. In a typical pregnancy, maternal tissues gradually become more insensitive to insulin, resulting in a 50–60% decrease in insulin sensitivity as gestation progresses [47,48]. During the second half of pregnancy, when IR is at its peak, GDM is believed to develop when β-cells fail to adapt to the increasing demand for insulin [47]. Both women with and without GDM experience similar increases in insulin production during pregnancy; however, women with GDM start at a lower level. Consequently, the β-cell abnormality in GDM is considered more of a chronic condition than a development during pregnancy. Overt T2DM may develop postpartum as a result of this β-cell malfunction in GDM [49]. Prior to and independently of changes in insulin sensitivity, the insulin secretory response significantly increases in the early stages of pregnancy. This metabolic adaptation may be mediated by circulating hormones, with placental hormones and/or cytokines likely responsible for these changes in maternal physiology [50].

Mostly women with preexisting IR and predisposing risk factors develop GDM. These risk factors include PCOS, low- or high-birth-weight fetuses, a family history of T2DM or GDM, multiparity, advanced maternal age, and a prior GDM diagnosis [51]. However, women with no preexisting IR may also develop GDM because they have defective β-cell function due to genetic or idiopathic reasons. IR in early pregnancy and a positive glucose challenge test in later pregnancy, irrespective of BMI, were also found to be associated with visceral adipose tissue depth evaluated by ultrasound that exceeded the top quartile in early pregnancy [52].

#### 3.1.1. Gestational Weight Status

Maternal weight is a crucial factor that significantly influences pregnancy progression, in conjunction with maternal age. Extensive studies have examined weight, calculating BMI in early pregnancy and the percentage of weight gained during pregnancy. Being overweight and obese are established contributors to GDM. A high antenatal BMI and excessive weight gain during pregnancy are recognized risk factors for various complications, including postpartum weight retention, GDM in subsequent pregnancies, future obesity, T2DM, and long-term CVD [53,54]. Additionally, these factors contribute to obstetrical complications such as pre-eclampsia, eclampsia, macrosomia, hemorrhage, and cesarean delivery [55]. Moreover, a higher maternal BMI is associated with an increased risk of perinatal mortality [56]. Weight gain during pregnancy is also linked to functional impacts on maternal glucose metabolism, particularly in obese and overweight women. While there are no changes in insulin secretion or clearance, there is a notable increase in IR [57].

Regarding maternal body fat levels, a significant correlation with maternal leptin levels during pregnancy has been observed. Leptin, a hormone produced by adipose tissue, has various metabolic effects, including decreased insulin sensitivity in non-pregnant individuals [58]. Mothers with elevated body fat levels are also more likely to give birth to newborns who have a higher likelihood of being obese in adulthood [59]. Visceral adipose tissue (VAT) in the abdomen is associated with metabolic syndrome, IR, and an elevated risk of CVD in the future [60,61].

The detection of IR and glucose dysregulation in mid-pregnancy is correlated with an increased depth of VAT in the first trimester of pregnancy [25,37]. According to a study by Rocha et al., VAT measured by ultrasound, placed from the aortic anterior wall to the linea alba, during the first half of pregnancy can predict the occurrence of GDM during the third trimester, even in non-obese pregnant women. VAT could thus be used as an accurate marker for GDM, regardless of BMI [56].

Specific recommendations now exist regarding the proportion of weight gain during pregnancy based on the woman’s BMI to prevent complications for both the mother and the fetus [62,63].

#### 3.1.2. Effects of Diet on Gestational IR

Diet, encompassing total calorie consumption, dietary plans, food processing, and preparation, as well as exposure to endocrine-disrupting chemicals [64,65], appears to exert a significant impact on the onset or exacerbation of IR, particularly in cases where IR already exists, such as in obesity or PCOS [66]. Systemic inflammation and IR are believed to be influenced by the quality and content of dietary fibers [67], while studies also indicate that diets rich in sucrose or fructose negatively affect IR [68].

A high-fat, Western-style diet characterized by a high caloric intake is a major risk factor for developing IR, prediabetes, T2DM, and obesity [69]. A diet rich in fats or sugars may also significantly alter the diversity of intestinal microbial flora. However, most diet-related alterations to the gut microbiota appear to be reversible with appropriate dietary modifications [70].

The diet recommended for expectant women with GDM should aim to promote healthy maternal weight gain while minimizing postprandial glucose spikes and fostering fetal development. Various dietary recommendations for GDM have been compared in numerous studies, including energy-restricted versus unrestricted diets; low-CHO, -GI, and -GL diets versus high-CHO, -GI, and -GL diets; diets rich in monounsaturated fats versus high-CHO diets [71]; and conventional diets versus fiber-enriched diets.

Recent research underscores the significance of replacing simple CHOs with complex CHOs rich in dietary fiber, while limiting the intake of simple sugars, especially from sweet drinks and treats, and avoiding excessive fruit juice consumption [72]. The inclusion of whole grains, substantial amounts of non-starchy vegetables, and fruits is crucial in the diet of individuals with IR [72]. A suitable diet for individuals who are insulin-resistant should also emphasize gradual and mindful eating, according to numerous researchers [72]. Additionally, chrono-nutrition and sleep hygiene appear to have a significant impact on the dietary habits of women with GDM [73]. Postprandial insulin responses have been shown to be influenced by the endogenous circadian (24 h) rhythm and metabolism, affecting various functions from intracellular biochemistry to whole-organism physiology [73,74]. However, the optimal diet remains a topic of ongoing debate.

#### 3.1.3. Effects of CHO and GI/GL Estimates on Gestational IR

Women with gestational GDM should exercise extra caution regarding both the quantity and type of CHOs they consume. All pregnant women require a minimum of 175 g of dietary CHOs, 71 g of protein, and 28 g of fiber daily, as CHOs serve as a vital energy source for both the mother and the fetus [75]. The nutrition plan should highlight monounsaturated and polyunsaturated fats while limiting saturated fats and avoiding trans fats [21].

A low-CHO diet is typically recommended for women with GDM, despite inconsistent findings in several studies [76]. Especially for women with T1DM, optimal metabolic regulation and more successful pregnancies seem to be achieved through CHO counting [77]. A moderately low-CHO diet comprising 40% of the recommended daily calories improves glycemic management in women with T2DM but does not demonstrate beneficial effects on pregnancy outcomes [78]. While a low-CHO diet improves short-term glycemic control in women with GDM, no impact on insulin requirements (in women receiving insulin treatment) or the success of pregnancies has been observed [50,79]. However, caution is advised when combining a low-CHO dietary pattern with a high consumption of animal-based protein and fat, as it appears to be associated with a higher risk of GDM and T2DM later in life [44,79]. Hernandez et al. also suggested that contrary to conventional advice, a high-complex-CHO/low-fat diet may improve maternal IR and reduce newborn obesity based on a dietary intervention pilot study [80].

A high GL diet has been associated with an increased risk of GDM in women, particularly when compared to those with the lowest tertile of dietary GL [81]. Younger gestational ages, higher CHO proportions, and lower fiber intake were strongly linked to high-GL diets [19]. In addition, a high-GI diet has been associated with elevated triglyceride levels [82].

Diets with a low GI and GL, such as the DASH diet and the Mediterranean diet, have demonstrated positive effects on various biochemical and health parameters [67,72,83]. Regarding obstetric and fetal outcomes in GDM patients, lower-GI diets have demonstrated potential benefits, although the findings are still debatable [81]. Notably, lower insulin utilization was observed in individuals adhering to a low-GI diet, characterized by the consumption of high-quality, complex CHOs [18,84,85]. Additionally, a low-GI diet showcased a capacity to reduce post-meal blood glucose levels in healthy individuals [20] and enhance lipid profiles in patients with GDM [86,87]. Moreover, women with normal glucose tolerance, GDM, or T2DM may experience less maternal weight gain when following a low-GI diet [18,52]. This dietary approach has also been linked to reduced glucose swings and decreased inflammatory markers, as evidenced by lower C-reactive protein levels [18]. Notably, a low-GI diet emerged as the most suitable dietary intervention for GDM patients, correlating once again with a lower frequency of insulin use [66,88].

Examining the impact on offspring, low-GI diets were associated with a decreased incidence of large-for-gestational-age (LGA) babies [85], as well as influences on birth length and early childhood arterial wall thickness [89]. The Homeostatic Model Assessment for Insulin Resistance (HOMA-IR), insulin, and leptin levels in children were also significantly were also significantly and positively correlatedwith dietary GI during pregnancy [90].

Contrary to these findings, several studies suggested that GI and GL indices were not significantly associated with GDM risk [26,91]. In patients with T2DM, Ojo et al. reported no notable differences in total cholesterol, HDL cholesterol, or LDL cholesterol [82]. Additionally, no distinctions were found in lipids [18,25], fructosamine, glycosylated hemoglobin [18,25], overall glycemic control, or pregnancy outcomes in women with GDM. Tieu et al. found no discernible changes in the risk of GDM or LGA prenatal births, cesarean deliveries, or gestational weight gain between low- and moderate-to-high-GI dietary groups [7,19]. Despite a successful reduction in dietary GL, the UK Pregnancy Better Eating and Activity Trial (UPBEAT) intervention, a theoretically based intervention in obese pregnant women, did not reduce the risk of GDM in women or the frequency of LGA infant births [92]. Several other studies failed to report any substantial influence of low-GI diets on birth weight, birth weight centile, the prevalence of macrosomia, adverse pregnancy outcomes [93], or a baby’s development pattern throughout the first year of life [18].

### 3.2. Inflammation in Gestation

Low-grade inflammation induced by cytokines is a typical feature of pregnancy, playing a vital role in the finely controlled inflammatory response crucial for the development of placentation from implantation to labor [94]. Maternal obesity and GDM are strong risk factors for persistent low-grade inflammation.

Proinflammatory cytokines have been consistently linked to inflammation induced by obesity, showing higher levels in affected individuals [90]. In pregnancies affected by obesity, the placenta may undergo changes in shape and function as an adaptive response, acting as both a target and a source of inflammatory cytokines [94]. Adipose tissue also produces several inflammatory factors that regulate hunger and fat synthesis. These pro-inflammatory mediators play a role in the development of IR, overt diabetes mellitus, and other complications related to obesity [95,96,97,98].

Regardless of maternal BMI, specific inflammatory markers have been found elevated during pregnancy in women with a history of GDM and those who later develop GDM. Circulating tumor necrosis factor-alpha (TNF-α), as a biomarker of inflammation, decreases in early pregnancy, accompanied by an increase in insulin sensitivity [40]. In a study by Challier et al., obese women exhibited a 2–3-fold increase in placental macrophages along with elevated mRNA expressions of interleukin-1 (IL-1), TNF-α, and interleukin-6 (IL-6) [73]. While lean, overweight, and obese women show similar patterns of cytokine changes, those with a higher BMI tend to exhibit an increase in specific inflammatory markers (CRP and IL-6), although not consistently across all markers [94]. Serum C-reactive protein (CRP) levels during the late second and early third trimesters are associated with GDM and weight gain, whereas elevated highly sensitive CRP levels in GDM patients may indicate an increased risk of later developing T2DM [99].

#### Effects of Diet, CHOs, and GI/GL Indices on Gestational Inflammation

Subclinical inflammation during pregnancy is influenced by dietary patterns [100]. A high intake of saturated fats can induce inflammation and endothelial dysfunction, disrupting insulin signaling [68].

In women with a previous history of GDM, macronutrient intake, particularly diets low in protein, high in cholesterol, and rich in monounsaturated fatty acids, significantly contributes to gut microbial dysbiosis. This imbalance is linked to obesity, low-grade inflammation, and inadequate glycemic control. Therefore, modifying dietary habits to alter the gut microbiota composition could be a promising strategy for preventing T2DM in this population [42]. Notably, micronutrients and polyunsaturated fats, especially those found in fish and seafood, exhibit anti-inflammatory properties and are associated with a reduced risk of GDM [75].

Regarding specific inflammatory markers, a low-GI diet significantly reduces IL-6 in diabetic women compared to that in a higher-GI diet [82]. According to the ROLO study, a randomized control trial involving 621 individuals, leptin and inflammation-related biomarkers are not significantly affected by a low-GI diet during pregnancy. However, individuals adhering to low-GI recommendations exhibit a diminished response to the typical increase in IR observed in pregnancy with advancing gestation [52]. Undoubtedly, further research is needed in this area.

## 4. Postpartum Period and Long-Term Management

Following delivery, women with GDM who were not previously diagnosed as diabetic (with any type of DM, including T1DM, T2DM, MODY, and LADA) should discontinue any treatment if needed. The recommended timeframe for glycemic reassessment is six to thirteen weeks post-delivery [101], to decide if initiation of drug treatment is indicated. This is particularly advised if future pregnancies are planned.

Healthcare providers should educate patients about the long-term risks of both T2DM and GDM during the postpartum period. Both the American Diabetes Association (ADA) [102] and the National Institute for Health and Care Excellence (NICE) [103] recommend lifelong, annual glucose level evaluations. Various risk factors, such as family history, a history of GDM in previous pregnancies, and the need for insulin or oral glucose-lowering medications during pregnancy, influence the frequency of T2DM occurrence [101]. The risk for type 2 diabetes among women with GDM is 10 times greater than for women with a normoglycemic pregnancy [104]. Postpartum women with obesity are also more susceptible to depression, venous thromboembolism, and challenges related to breastfeeding [105].

Lifestyle modifications play a crucial role, especially for morbidly obese women with IR, prior GDM, or prediabetes. A diet rich in protein and regular exercise are strongly recommended, as they have proven to be more successful in reducing IR and improving glycemic variability [106]. Diets such as the Mediterranean and DASH diets, low in complex CHOs and GI/GL, particularly when combined with plant-based proteins and fats [44,45], are employed to reduce the likelihood of GDM in future pregnancies and the risk of developing T2DM.

## 5. Conclusions

The use of GI and GL estimates in the management of GDM has gained attention, recognizing the substantial impact of diet on glycemic control and pregnancy outcomes for GDM patients. Research indicates that low-CHO diets, particularly in the short term, have proven beneficial for patients with T2DM. Considering that women with GDM typically have diabetes for short durations (less than six months), low-CHO/low-GI diets may also be advantageous for them. A low-GI diet, in contrast to one with a higher CHO content, has been associated with improved glycemic control, reduced insulin requirements, lower cholesterol levels, decreased inflammation markers, and enhanced obstetric outcomes.

However, the data remain contradictory, with several studies reporting no significant associations. The limited number of studies examining the impact of GI and GL on GDM, the absence of a consensus on what constitutes a preferred GL/GI diet, and ethical constraints preventing the study of effects on infants and newborns except through observational studies hinder our ability to fully assess these diets’ effects. Until larger-scale intervention trials are conducted, a low-GI diet should not replace the current pregnancy diets recommended by health organizations. Achieving a general consensus on what constitutes a low-GI/-GL diet is essential to enhance clinical practice with specific dietary recommendations for GDM patients.

## 6. Future Directions

There is an urgent need for expanded nutrition research to enhance our understanding of precise dietary strategies for effectively managing GDM and improving glycemic control. It is crucial to explore various elements of the ideal diet for GDM patients, including the specific types of macronutrients and the necessity of particular micronutrients. Additionally, it is vital to determine whether changes in the quality of dietary CHO correspond to glycemic control in GDM, paralleling glycemic control in pregnant non-diabetic women.

Exploring whether there is a critical window for dietary CHO consumption, such as intake before pregnancy or throughout the two trimesters, is essential for potential GDM prevention. Subsequent studies could investigate the long-term effects of low-GI and -GL diets on hormonal status, maintaining a healthy weight before pregnancy in populations at high risk for GDM (such as obese, insulin-resistant, or PCOS patients) and preventing complications in the first trimester. Through additional research in this area, we can refine the dietary recommendations and develop unique management strategies for GDM that leverage the potential benefits of the GI and GL.

## Figures and Tables

**Table 1:** Representative foods low in GI (<55) and GL (<10).

Food	GI	GL	Food	GI	GL
Apples	40	6	Barley	28	13
Oranges	40	4	Rice noodles	54	22
Cherries	20	5	Full-fat milk	27	3
Raspberries	32	3	Low-fat milk	32	4
Grapefruits	25	3	Soy milk	55	6
Kiwis	53	9	Skim milk	32	4
Dates	54	21	Yogurt	15	1
Pear	33	3	Chocolate	43	7
Apricot	34	3	Fructose	23	2
Apple juice	40	12	Peanuts	14	1
Orange juice	55	14	Walnuts	20	1
Chickpeas	28	8	Almonds	10	<1
Kidney beans	28	7	Pecans	10	<1
Lentils	30	11	Hazelnuts	15	<1
Soybeans	16	9	Eggplant	15	2
Mushrooms	10	1	Celery	15	1
Zucchini	15	1	Spinach	15	1
Asparagus	32	2	Carrots	39	2
Cucumber	15	0	Tomatoes	15	1
Onion	15	<1	Lettuce	15	1

## Data Availability

Data are contained within the article.

## List of Abbreviations

ADA	American Diabetes Association
AGEs	Advanced glycation end-products
BMI	Body mass index
CHO	Carbohydrates
CRP	C-reactive protein
CVD	Cardiovascular disease
GDM	Gestational diabetes mellitus
GI	Glycemic index
GL	Glycemic load
HOMA-IR	Homeostatic Model Assessment for Insulin Resistance
IL	Interleukin
IR	Insulin resistance
LGA	Large for gestational age
miRNAs	Micro RNAs
ncRNA	Non-coding ribonucleic acid
NICE	National Institute for Health and Care Excellence
PCOS	Polycystic ovary syndrome
T1DM	Type 1 diabetes mellitus
T2DM	Type 2 diabetes mellitus
TNF-α	Tumor necrosis factor-alpha
VAT	Visceral adipose tissue
